# Microsatellites in Pursuit of Microbial Genome Evolution

**DOI:** 10.3389/fmicb.2015.01462

**Published:** 2016-01-05

**Authors:** Abdullah F. Saeed, Rongzhi Wang, Shihua Wang

**Affiliations:** Key Laboratory of Biopesticide and Chemical Biology of Education Ministry, School of Life Sciences, Fujian Agriculture and Forestry UniversityFuzhou, China

**Keywords:** microsatellites, short sequence repeats, DNA sequences, genetics, evolution, molecular, microbiology

## Abstract

Microsatellites or short sequence repeats are widespread genetic markers which are hypermutable 1–6 bp long short nucleotide motifs. Significantly, their applications in genetics are extensive due to their ceaseless mutational degree, widespread length variations and hypermutability skills. These features make them useful in determining the driving forces of evolution by using powerful molecular techniques. Consequently, revealing important questions, for example, what is the significance of these abundant sequences in DNA, what are their roles in genomic evolution? The answers of these important questions are hidden in the ways these short motifs contributed in altering the microbial genomes since the origin of life. Even though their size ranges from 1 –to- 6 bases, these repeats are becoming one of the most popular genetic probes in determining their associations and phylogenetic relationships in closely related genomes. Currently, they have been widely used in molecular genetics, biotechnology and evolutionary biology. However, due to limited knowledge; there is a significant gap in research and lack of information concerning hypermutational mechanisms. These mechanisms play a key role in microsatellite loci point mutations and phase variations. This review will extend the understandings of impacts and contributions of microsatellite in genomic evolution and their universal applications in microbiology.

## Introduction

Microsatellites or simple sequence repeats (SSRs) are short tandem repeats (STRs) of DNA sequence motifs predominantly abundant in various genomes and have been widely used for genetic studies and molecular markers (Han et al., 2015). The term “microsatellites” was first coined in by Litt and Luty (1989) during their work on (TG)n in gene of cardiac actin. These repeats were developed for the study of neurological diseases in human and afterward their applications made them significant in various molecular fields.

They are also known as single nucleotide polymorphisms (SNPs) (Batley et al., 2007), are associated and located at telomeres, centromeres, genic regions, intergenic regions and even at interspersed sites within a genome (Kim et al., 2015). SSRs are named as the most versatile molecular markers used to identify a certain molecular sequence in a pool of unknown DNA; they have applications in various fields of molecular biology, biotechnology and evolutionary biology. Tandem repeats are ubiquitous and widely used in genetic studies of microbes (Grover and Sharma, 2012; Poczai et al., 2013; Abdul-Muneer, 2014; Zhao et al., 2015). These markers are principal tools of determining hyper mutational genetic diversity by recently developed advanced sequence techniques in genetics (McCouch et al., 2002).

DNA is a polymorphic molecule, extremely stable in hostile environments and accountable for the inheritance of traits through generations by conserving genetic code of the host organism (Gyllensten et al., 1991; McKusick, 1998; Shitara et al., 1998; Birky, 2001). It has been demonstrated that SSR markers are repeated frequently in a conserved DNA sequence and suitable for studying genetic diversity among species, populations, and individuals. Various techniques have been established to evaluate DNA polymorphism by measuring genetic diversity *in situ*. Consequently, it is easy to trace the fingerprints of all the organisms by examining molecular markers of DNA involved in determining the inherited characters and evolutionary history in a phyletic lineage (McCouch et al., 1997, 2002).

The difference in number of repeats of SSR motifs in different species shows polymorphism (Xu et al., 2012). Low DNA amounts are needed for the amplification of genomic DNA; therefore, SSRs are polymerase chain reaction (PCR-based) markers and mostly co-dominant, multi-allelic, reproducible, and highly polymorphic (Birren and Lai, 1987; Powell et al., 1996b; Feingold et al., 2005). Generally, they have been used in genic linkage mapping, genetic characterization of germplasmic resource investigation, phylogenetic analysis, DNA fingerprinting and other genetic studies (Liu et al., 1996; Pérez et al., 2005; Weng et al., 2007). Satellite DNAs are generally related with centromeric heterochromatin and are being progressively employed as a useful tool for genome analysis, mapping and for understanding chromosomal organization (Megan et al., 2014; Shen et al., 2015). They are used for genome mapping, population studies, and specie identification and continued to be the genetic marker of choice in most non-human systems and form an important genomic component (Amos et al., 2015).

Microsatellites are characterized by tandemly repeated short motifs with length 1 –to- 6 bp long core sequences. Their hypervariability is based on changes in the repeats of core sequences several times at a given locus (Tautz, 1993; Varshney et al., 2005; Zulini et al., 2005; Wheeler et al., 2014). They can be traced in both coding and non-coding regions (Ellegren, 2004). Generally, there are three classes of biological markers: (i) nucleic acid hybridization, e.g., restriction fragment length polymorphisms (RFLPs) (ii) PCR-based on DNA amplification, e.g., random amplification of polymorphic DNAs (RAPD), amplified fragment length polymorphisms (AFLP), SSRs and (iii), SNPs (Priyono and Putranto, 2014; Vijay et al., 2015).

Molecular biology became progressive and innovative with the invention of PCR technology in mid 1980s (Saiki et al., 1985; Mullis and Faloona, 1987), this revolutionary technique facilitated in various biological fields, i.e., diagnostics, breeding programs, forensics, microbiology etc. Consequently, microsatellite maker systems are widely used in evolutionary biology due to their hypervariability and hypermutability (Dallas, 1992; Weber and Wong, 1993; Di Rienzo et al., 1994; Ellegren, 1995). Microsatellites are tandem repeated motifs of variable lengths found throughout cellular nuclear genomes (Jarne and Lagoda, 1996). They also appear in organelle genomes, e.g., chloroplast (Powell et al., 1995, 1996a) and mitochondria which were predominantly widespread in primitive microbial world (Soranzo et al., 1999). It is convenient to genotype microsatellites instead of their polymorphic variability nature, because, they are densely populated throughout genomes. Therefore, they are useful genetic markers in high resolution genetic mapping (Dib et al., 1996; Dietrich et al., 1996; Schuler et al., 1996; Knapik et al., 1998; Cooper et al., 1999).

In 1986, the role of microsatellites in microbial DNA was identified in *Neisseria gonorrhoeae*; a bacterium which is responsible for infamous sexually transmitted disease (STD) gonorrhea. This bacterium possesses family of 12 outer-membrane proteins which are encoded by *Opas* genes. These proteins upon expression help bacterium to adhere invading epithelial cells. The *Opas* genes retain multiple copies of microsatellites comprising of 5 based motif CTCTT (Moxon et al., 1994; Hung and Christodoulides, 2013). Several SSRs have been identified with their physiological and morphological functions in microbial genomes as shown in **Table 1** (Peak et al., 1996; Burch et al., 1997; Inzana et al., 1997; Karlin et al., 1997; Grimwood et al., 2001; Rocha and Blanchard, 2002).

**Table 1:** Microbial coding regions containing simple sequence repeat (SSRs), physiological and morphological effects in various species.

Related species	Repeat sequence(s)	Associated gene	Physiological and morphological functions
*H. influenzae*	CAAT	Virulence gene	Adaptational phase variation
*H. somnus*	CAAT	Virulence gene	Lipooligosaccharide (LOS) phase variation
*Neisseria* sp.	GCAA	Virulence gene	Adaptational phase variation
*M. catarrhalis*	CAAC	Virulence gene	Adaptational phase variation
*M. hyorhinis*	AGT	Lipoprotein gene	Genomic translational regulation
*N. gonorrhoeae*	(G)_n_	LOS gene	LOS-specific Morphological variation
*Chlamydia pneumoniaes*	(G)_n_ (C)_n_	Membrane protein gene	Elicits contagious cellular pathogenesis

Microsatellites are the most useful molecular markers with an advantage of easy and low-cost detection by PCR due to high mutation rates and new sequencing technologies. Therefore, their applications in microbiology are widespread for the determination of genomic evolution (Paglia and Morgante, 1998). As compared to RAPD and AFLP, which can detect the location of locus in a genome, microsatellites have an advantage, because, they can detect all the physiological parameters of a genome (Maughan et al., 1996; Powell et al., 1996b; Beismann et al., 1997; Gaiotto et al., 1997; Russell et al., 1997; Witsenboer et al., 1997; Semblat et al., 1998; Nybom et al., 2014). The present study aimed to investigate the roles of microsatellites in shaping the genomes over time and to develop better understandings of their characteristic hypermutability and hypervariability by employing advanced molecular techniques. This will help extend substantial knowledge about their significant importance in genome evolution.

## The Origin and Frequency of Microsatellites

### Origin

The origin of microsatellites in microbial genomes is non-random, with various differences among the mechanisms which stimulated for SSRs genes, these mechanisms consisted of insertions, deletions, recombination and repair, transpositions, horizontal gene transfer and replication slippage (Hancock and Santibáñez-Koref, 1998; Primmer and Ellegren, 1998; Alba et al., 1999, 2001; Chambers and MacAvoy, 2000; Hartenstine et al., 2000; Jakupciak and Wells, 2000; Schlotterer, 2000; Zhu et al., 2000; Bhargava and Fuentes, 2010; Holder et al., 2015).

Currently, there are two non-mutually special hypotheses to describe the source of microsatellites: (i) *De novo* theory suggests that the microsatellites originated from a proto-microsatellite in microbes, a small region of as few as three or four repetitive elements within simple sequences, which are distinct as a struggle of repetitive motifs deficient in clear tandem organization (Messier et al., 1996; Buschiazzo and Gemmell, 2006; Wang et al., 2015). Consequently after formation, the conservation and proliferation was selected by strand slippage through replication and subjected to the repeat motif, it had a capacity to form unusual DNA conformations and contributed in recombination and transposition events. The number of repeat units runs parallel with the variability of microsatellite, but the least repeat number which is significant for strand slippage and other mutations is uncertain (Jentzsch et al., 2008). Slippage mutations occur repeatedly at runs of 3–4 bases in prokaryotic genomes (Foster and Trimarchi, 1994; Rosenberg et al., 1994; Sébastien et al., 2010).

(ii) Adopted microsatellites theory suggests their beginning from other genomic sections via transposable elements. The transposable elements consisted of one or more locations susceptible to microsatellite development and favored the distribution of microsatellites in genomes. This advocates a mutual association in which microsatellites acted as “retroposition navigator sequences,” while retrotransposons produced more microsatellites during their scattering in genomes. An example of a retrotransposon-mediated microsatellite birth is the origin of A/T rich microsatellites with motifs extending from 1 to 6 bases in length from Alu elements (Wilder and Hollocher, 2001; Jentzsch et al., 2008; Sand et al., 2015).

### Frequency and Classification

Microsatellites are DNA sequences of 1–6 bp units repeated in tandem and widely dispersed in the microbial genome (Powell et al., 1996a). Numerous repetitive sequences including microsatellites are found in up to 5% of the prokaryotic DNA (Ussery et al., 2004; Wheeler et al., 2014). The frequency and spreading of SSR is centered on species and motif specificity (Karlin et al., 1996, 1997; Bachtrog et al., 1999; Butcher et al., 2000; Crollius et al., 2000; Metzgar et al., 2000; Tóth et al., 2000; Gentles and Karlin, 2001; Morgante et al., 2002; Katoh et al., 2015). SSRs with 1–6 bp were used for phase variation in bacterial adaptations (Holder et al., 2015).

Microsatellites can be amplified with the help of PCR in rigid conditions with the amplification of single loci (Bravo et al., 2006; Buschiazzo and Gemmell, 2006). They are broadly distributed in various genomes and highly polymorphic in nature. Therefore, establishes the foundation of their success in wide range of biological fields (Chistiakov et al., 2006).

Simple sequence repeats in various organisms are also noticeable from the diverse genome regions, e.g., 3′-UTRs, 5′-UTRs, exons and introns (Rajendrakumar et al., 2007). Their localization can be altered by different aspects of DNA structures (Chistiakov et al., 2006). The transposable elements help in the formation and dispersion of microsatellite throughout the genome (Bhargava and Fuentes, 2010). Kashi et al. (1997) described the length of SSRs which influences the transcriptional activity in promoter regions.

The effect of length variations in the mononucleotide repeats and polymorphisms within these regions of chloroplast genome are used to study both intraspecific and interspecific variability (Powell et al., 1995). Length variation at a mitochondrial SSR locus was first reported by Soranzo et al. (1999). The descriptive analysis of microsatellite content in genome sequences reflects their roles in genome organization, recombination, gene regulation, quantitative genetic variation and gene evolution (Katti et al., 2001).

**FIGURE 1:**
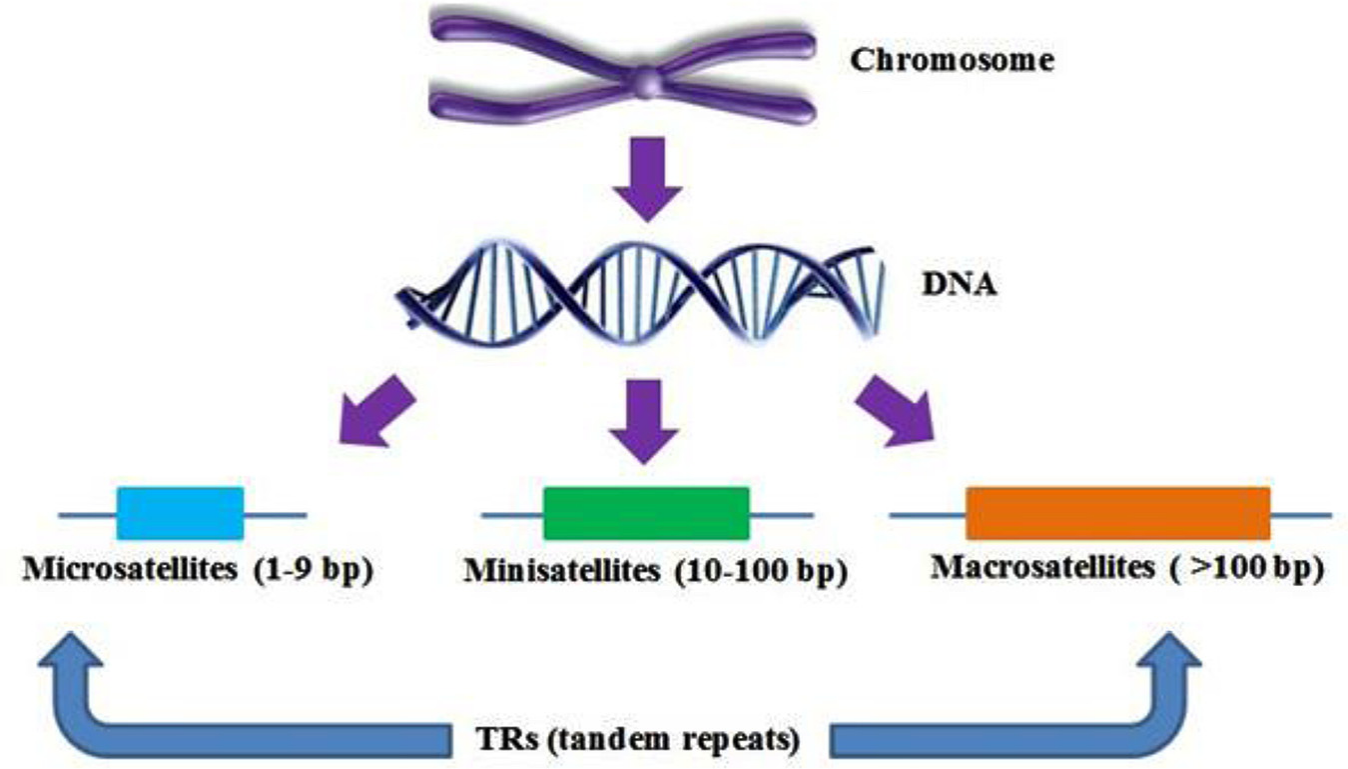
**Diagram illustrating the different types of tandem repeats (TRs).** The width of boxes has been shown to develop visual precision of the figure.

Classification of SSRs is based on their isolation and sequencing. They have variable length of repeat motifs from just a single base to thousands of bases; microsatellites can be classified on the number of bases, i.e., short repeats (10–30 bases) known as minisatellites and with longer repeats (between 10 and 100 bases) are called macrosatellites, satellites with even shorter repeat motifs, called microsatellites (**Figure 1**). Based on the length of the repeat units, SSRs are categorized into three groups (Class I>20 bp, Class II=between 11 and 20 bp, and Class III<11 bp), Scattered repetitive elements are determined at the flanking sites of the SSRs. (Temnykh et al., 2001; Varshney et al., 2002).

Abundance and length variations in microsatellites motifs are evaluated on mono, di, tri, tetra, penta and hexanucleotide. (Rabello et al., 2005; Merritt et al., 2015). They are also classified according to the type of repeated sequence presented: (i) perfect repeats, with perfect repetitions, e.g., (AT)20, have sequences of ten or more mononucleotide repeats, six or more dinucleotide repeats, tri, tetra and pentanucleotide repeats. (ii) Imperfect repeats, with interruption by different nucleotides which are usually not repeated, e.g., (AT)12 GC(AT)8, and (iii) composite, with two or more different motifs in tandem, e.g., (AT)7(GC)6. FORESTs database showed complementary sequences belonging to the same class (e.g., AC, CA, TG, GT). (Temnykh et al., 2001; Selkoe and Toonen, 2006). Compound microsatellites are present in the same expressed sequence tag (EST) at a distance by a maximum of 100 bp. A repeat having more than 50 bp distance from the 3′ end of sequences is not considered as microsatellite (Rabello et al., 2005; Vogiatzi et al., 2011; Wang et al., 2015).

*Lactobacillus* species revealed a wealth of compound imperfect microsatellites clustered in the coding regions of genomes. They were consisted of variant motifs with maximum distance (*d*_MAX_) increments of 10–50. The variations analyzed in compound microsatellite of *Escherichia coli*, and lactobacilli suggested diverse genomic features and evolutionary traces of compound microsatellites between these organisms (Basharat and Yasmin, 2015).

## Occurrence of Microsatellites in Genomic Evolution

Simple sequence repeats like microsatellites are found abundantly in prokaryotic genomes, these repeats are extremely important molecular markers for the investigation of population genetics of genomes on the bases of excessive polymorphism, reproductivity, and codominance (Field and Wills, 1998; Schlotterer, 2000). 1,117 microsatellite patterns in about 3.8 Mb of unique sequences (0.47% of the total DNA used in the analysis) were identified in *Paracoccidioides brasiliensis*. 87.5% microsatellites were found in non-coding sequences (Nascimento et al., 2004). The applications of SSRs in genomic studies on molecular basis (Jarne and Lagoda, 1996), their evaluation of population dissemination and evolutionary relationships (Queller et al., 1993), have been used frequently in the study of parentage investigation, phylogenetic studies (Bowcock et al., 1994), studies on population diversity (Paetkau, 1999), determination of inbreeding (Coltman et al., 1998; Coulson et al., 1998), genetic recombinations, population genetic assembly, genomic mapping, and phylogeography (Sunnucks, 2000; Zhang and Hewitt, 2003).

Microsatellites are significant in evaluation of the ability of individual migrations, resemblances in vast extent of the organisms, ranging from mammals and higher chordates to lower microbes such as fungi and even prokaryotes and viruses (Ashley and Dow, 1994; Dib et al., 1996; Selkoe and Toonen, 2006; Breurec et al., 2011).

Debatable hypothesis had been confronted by the genetic evidences came from microsatellites like famous hypothesis put forth by Bass-Becking, “Everything is everywhere, the environment selects” (Baas Becking, 1934). These repeats are vital to differentiate morphologically different species on molecular basis. (Katz et al., 2005). Hatcher et al. (2015) reported that the poxvirus genomes consisted of 24% microsatellites nucleotide sequences. They exhibit hypervariations in poxvirus proteins, gene truncation, and reductive evolution. They are also widely used in the fields of genomic mapping, sex determination, environmental resource and genetics, evolutionary lineages of microbial strains and analysis of phylogenetic relationships in closely related species (Jarne and Lagoda, 1996; Hennequin et al., 2001; Luikart et al., 2003; Lim et al., 2004).

### *Escherichia coli* (ECOR)

To study the microbial evolution and phylogenetic relationships, *Escherichia coli* (*E. coli*) reference strains are significant and most often used in determining the evolutionary relationships among microbes (Ochman and Selander, 1984), Several *E. coli* strains have been classified into six phylogenetic groups (A, B1, B2, C, D, and E) on the bases of multilocus enzyme electrophoresis (MLEE) method (Goullet and Picard, 1989), most importantly, these strains do not make assemblies within distinct phylogeny on the bases of rep-PCR DNA fingerprinting arrays (Johnson and O’Bryan, 2000). Metzgar et al. (2001) also reported similar applications for utilization of microsatellites at a greater extent in evolutionary analyses to characterize microbial strains.

### Haemophilus influenzae (Hi)

Microsatellites are hypermutable in every generation, tetranucleotide repeats lose and gain units at a rate of 1 × 10^-4^ (De Bolle et al., 2000) suggesting that this high decline rate in prevalence reveals evolution by natural selection. Excessive rate of loci mutations results into harmful fitness effects rather than beneficial. SSRs are found abundantly in some host-adapted bacteria as compared to other genomes (Mrázek et al., 2007). It is shown that long tracts of tetranucleotide repeat sequences are abundantly found in the *Hi* strain Rd KW20 genome; these repeats have an association with the genes which control commensal and virulence behavior (Hood et al., 1996).

## Microsatellite Isolation, Identification and Sequencing Methods

### Isolation

To study microsatellites, several approaches have been established with the recent development of advanced molecular techniques. These protocols can be grouped into three types: (i) the standard method, where a library is screened (ii) the automated method, sequences are searched in sequence databases and (iii) the sequencing method, whole or parts of the genome are sequenced. These methods are modified and optimized on the bases of species and conditions (Zane et al., 2002; Weising et al., 2005).

### Identification of Microsatellites

In the 1960s, simple repeats were identified in density gradient centrifugations of randomly sheared genomic DNAs by way of a ‘satellite peak’ and found dispersed throughout various genomes (Park et al., 2009). Different techniques have been introduced to identify microsatellites (Dutech et al., 2007). The most common methods in used for the identification of repeats are the target enrichment of DNAs (Hamilton et al., 1999; Zane et al., 2002). One method being employed is known as inter simple sequence repeat PCR (ISSR-PCR), in which ISSR primers containing microsatellites motifs along with three anchored nucleotides at 5′ terminal end are used for amplifying microsatellite sequence regions which are known to be abundant in genomes, the PCR products are then cloned and sequenced for determination of microsatellites (Zietkiewicz et al., 1994; Van Der Nest et al., 2000).

With recent development in molecular biology, modifications in DNA enrichment strategies are made, linking hybridization with probes to identify and compare a vast range of microsatellite sequences to genomic DNA fragments (Zane et al., 2002). One of the current approaches being used is called fast isolation by AFLPs of sequences containing repeats (FIASCO), which follows amplified fragment length polymorphism (AFLP) (Vos et al., 1995) Both ISSR-PCR and FIASCO methods are routinely applied in studies related to the identification and characterization of SSRs and they have been used to isolate microsatellites from various microbial species (Luque et al., 2002; Squirrell et al., 2003; Pfunder and Frey, 2006; Barnes et al., 2008; Santana et al., 2009).

### Other Approaches for Microsatellite Identification

In advent of recent development in identification strategies of microsatellites, various methods have been devised for characterization of microsatellites.

#### Development of a Clone Library

One method is the development of a library with the help of various protocols to create and screen a cDNA or PCR fragment library, in this method the DNA is fragmented by sonication or enzymatic digestion, then fragments are ligated into a vector and transformed into *E. coli*, following clones are analyzed by southern blot for SSR and finally the positive clones are sequenced (Weising et al., 2005).

The positive clone obtained ranges from 0.04 to 12% (Zane et al., 2002). The plasmids of fragment library can be screened by the use of biotinylated oligonucleotides (Ito et al., 1992). In another method, the genomic library was amplified using biotinylated oligonucleotides complementary to SSRs, as primers (Paetkau, 1999). A high enrichment efficiency of almost 90% for CA repeats was generated by using two rounds of amplification and hybridization with biotin/streptavidin (Kandpal et al., 1994).

#### High-Tech Methods

Microsatellite identification and development can be done by using public DNA databases, such as BLASTN (Altschul et al., 1990; Dhillon et al., 2014). Various programs and reference lists are available in the database (Mittal and Dubey, 2009). Numerous studies have been used to search for more conserved and gene related microsatellites by using EST-SSRs (Varshney et al., 2005).

#### Sequencing Methods

Expressed or whole genome sequencing can be made by new high-tech sequencing techniques (Abdelkrim et al., 2009; Mikheyev et al., 2010). With the use of inconsistent PCR amplification, approximately half of all microsatellite loci are lost (Arthofer et al., 2011)

Microsatellite markers from microbial genomes of model and non-model organisms are being isolated by use of next generation sequencing (NGS) like Roche 454 GS-FLX Titanium pyrosequencing platform, this technique has a potential for the isolation of microsatellite markers from the genome of both model and non-model species with no former reference genome existing (Margulies et al., 2005; Malausa et al., 2011). Four hundred and fifty-four pyrosequencing has many proficient advantages over customary enrichment techniques in isolating microsatellite markers because of high throughput, cost effective, rapid and low labor supplies (Rothberg and Leamon, 2008).

Currently, a new technique Comparative genomic hybridization (CGH-style) array manufactured by Nimblgen/Roche has been used to rapidly measure the complete microsatellite content of a genome. CGH-microarray measures DNA samples labeled with different fluorescent dye from a reference genome and a test genome, and hybridizes them competitively to develop a micro-assay based array comprised of immobilized DNA fragments from sequence of the reference individual (Hazen and Kay, 2003; Hardiman, 2004; Dorrell et al., 2005; Fan et al., 2006).

This technique sums the contributions for a specific repeated motif from number of sites in which that particular motif exists across the whole genome. CGH-array has the ability to assess 1 -to- 6 mer repeats. This method provides significant information about genetic distances for entire genes between pairs of entities in one assay and has made CGH array an attractive tool for phylogenetic analysis. Numerous research approaches applied this microarray to compare the evolutionary relationships of bacterial species (Israel et al., 2001; Chan et al., 2003; Wolfgang et al., 2003; Rasmussen et al., 2008; Igboin et al., 2009; Dorrell et al., 2011).

Guidot et al. (2007), Wan et al. (2007), and Dagerhamn et al. (2008) reported applications of CGH array to recover clusters of bacteria from large clone libraries; it is parallel with formerly described MLSA phylogenies. Solheim et al. (2009) described comparison of MLST phylogeny with CGH array used for *Enteroccocus* species to define lineage-specific genes in entire reference genome.

Recently, NGS technologies is the most powerful method available to generate cost effective DNA markers including SSRs and SNPs. NGS technologies are integrated with tools like association mapping studies. The NGS method is far more powerful than any existing in generating DNA markers and dramatically increased the yield of potential microsatellite primer pairs, generating 1000s of individual reads (Ekblom and Galindo, 2011; Hoffman and Nichols, 2011; Castoe et al., 2012; Smulders et al., 2012; Yang et al., 2012; Lance et al., 2013; Andersen and Mills, 2014; Vukosavljev et al., 2015), the development of molecular markers is based on short-length sequences from genomic DNA sequences or cDNA (RNA-seq) (Yang and Smith, 2013).

## Determination, Hypermutability and Portability of SSRs Loci

### Determination

The analysis of loci is determined by the number of repeated motifs and on polymorphic level with specificity in population (Weising et al., 2005). Several statistical analysis based on genetic distances can be utilized along with the use of similarity index and band sharing data (Labate, 2000; Weising et al., 2005; Excoffier and Heckel, 2006). Excoffier and Heckel (2006) accredited two conversion programs for formatting input data files: Convert (Glaubitz, 2004) and Formatomatic (Manoukis, 2007).

### Genomic Evolution Through Hypermutability

Microsatellites are extremely hypermutable as associated with point mutations in coding, non-coding genes and mutation rates which range from 10^-6^ to 10^-2^ events per locus per microbial generation. These rates are greatly affected by numerous features, which affect both the likelihood of mutational generations and the restoration proficiency of these mutations (Jarne and Lagoda, 1996).

Evolution has operated on bacterial microsatellite loci at mutation rates up to 1 × 10^-3^ per division in combination with trans-acting factors; this mutability in bacterial pathogens is known as localized hypermutation. The mechanisms involved site-specific recombination, homologous recombination of tandem duplications of DNA sequences, SSR and G-quartet-mediated gene conversion in pilin sub-unit of *Neisseria*. This gave rise to specific phenotypes by presumptive, high frequency, reversible switches of associated gene expression. These switches are also responsible for phase variations observed in various bacterial genomes (Bidmos and Bayliss, 2014).

Mutation mechanisms, DNA healing, organizational features of microsatellite, genomic specific framework and selective biological impacts are important factors which relate and control the evolutionary dynamics of microsatellites. In prokaryotes, resilient progressive selective pressures are related with extremely mutable microsatellite loci stretches in genomes that regulate pathogenicity. The average mutation rate of a bacterial gene is 1 × 10^-9^ mutations/division, but mutation rates of microsatellites are significantly higher than this average. (Moxon et al., 1994; Bidmos and Bayliss, 2014). Large numbers of SSRs are supposed to evolve neutrally; the most extensively considered exclusions are the increasing number of triplet-repeat loci which are the source of genetic diseases (Sutherland and Richards, 1995). It is clear that the investigation of the evolutionary associations of tandem repeat sequences in microbial genomes with respect to genome volatility and utility is significantly supported by rapid emergence of many newly sequenced genomes (Strauss and Falkow, 1997).

### Portability of Microsatellites

Microsatellites are easily transferable to the related genomes which have high proportion of similar conserved transcribed domains (Cordeiro et al., 2001; Decroocq et al., 2003; Hempel and Peakall, 2003; Varshney et al., 2005). The detection of fractional polymorphism with these repeats showed high rates of portability within genomic regions (Cho et al., 2000; Scott et al., 2000; Eujayl et al., 2002), this ability is also associated with differences in gene expression rooted in various microbial species (Gao et al., 2004). Pandian et al. (2000) examined transferability of SSRs in many genomes and revealed a high level of sequence conservation.

The prevalence of flanking regions among microsatellites allows cross-species amplification (Rico et al., 1996; Peakall et al., 1998). Around 20 microsatellite markers are used for characterizing transferability and polymorphism by EST databases (Faria et al., 2010). Pépin et al. (1995) showed 40% of microsatellites are useful to study genomes of important loci. Dawson et al. (2010) developed primer sets from 33 polymorphic loci. The capacity of transferability can be determined by the extent of genomic sequence matching and by the use of interspecies sequence markers (Gupta et al., 2013).

## Genomic Evolution Through Mutations

### Point Mutations

Microsatellites constructed for specific species can be applied to other species closely related to each other. But if the genetic distance increases, the percentage of successful amplification of loci decreases (Jarne and Lagoda, 1996). “Null alleles” are formed with the occurrence of primer annealing point mutations and microsatellites fail to amplify the PCR product (Jarne and Lagoda, 1996; Dakin and Avise, 2004).

### Mechanisms of Length Variations

Microsatellites are tandemly repeated number of times. They are predominant genetic markers in molecular biology with DNA sequences of 1–6 bp in length. Essentially, the repeat-motifs containing more than mono-nucleotide are selected to develop molecular markers. To pursue a SSR, different parameters such as repeat sequence length, coding position, repeat category (mono- hexa), and sequence motifs are employed (Dikhit et al., 2014).

The molecular processes which expose DNA individual strands result in sequence repeat length mutations comprising of replication, recombination, DNA damage repair and rest of DNA metabolic processes (Wells et al., 2005; Lopez Castel et al., 2010). Microsatellite is prone to length mutations because of intrinsic features of repeat sequences such as unit length, number of repeats, and its structural purity (Fondon et al., 1998; Legendre et al., 2007). Mutation rates due to replication slippage at microsatellite loci are hypervariable extending from unnoticeable level to roughly about 8 × 10^-3^ (Mahtani and Willard, 1993; Weber and Wong, 1993; Strand et al., 1994; Tautz and Schlötterer, 1994).

Length changes in SSRs are occurred due to the replication slippage and loops because of mismatched DNA strands during replication, excluding *Helicobacter pylori* which has remarkably extended mono- dinucleotide repeats since they are physiologically functional (Tomb et al., 1997) or in case its genome lacks mismatch DNA repair (Eisen et al., 1997). Upon denaturation of daughter strand in replication, it will pair with wrong sequence complementary to the template strand and will result in sequence deletion or insertion. This type of microsatellite mutation occurs roughly once per 1,000 generations and are more prevalent than the point mutations in other genomic sites (Weber and Wong, 1993; Tautz and Schlötterer, 1994; Jarne and Lagoda, 1996).

**FIGURE 2:**
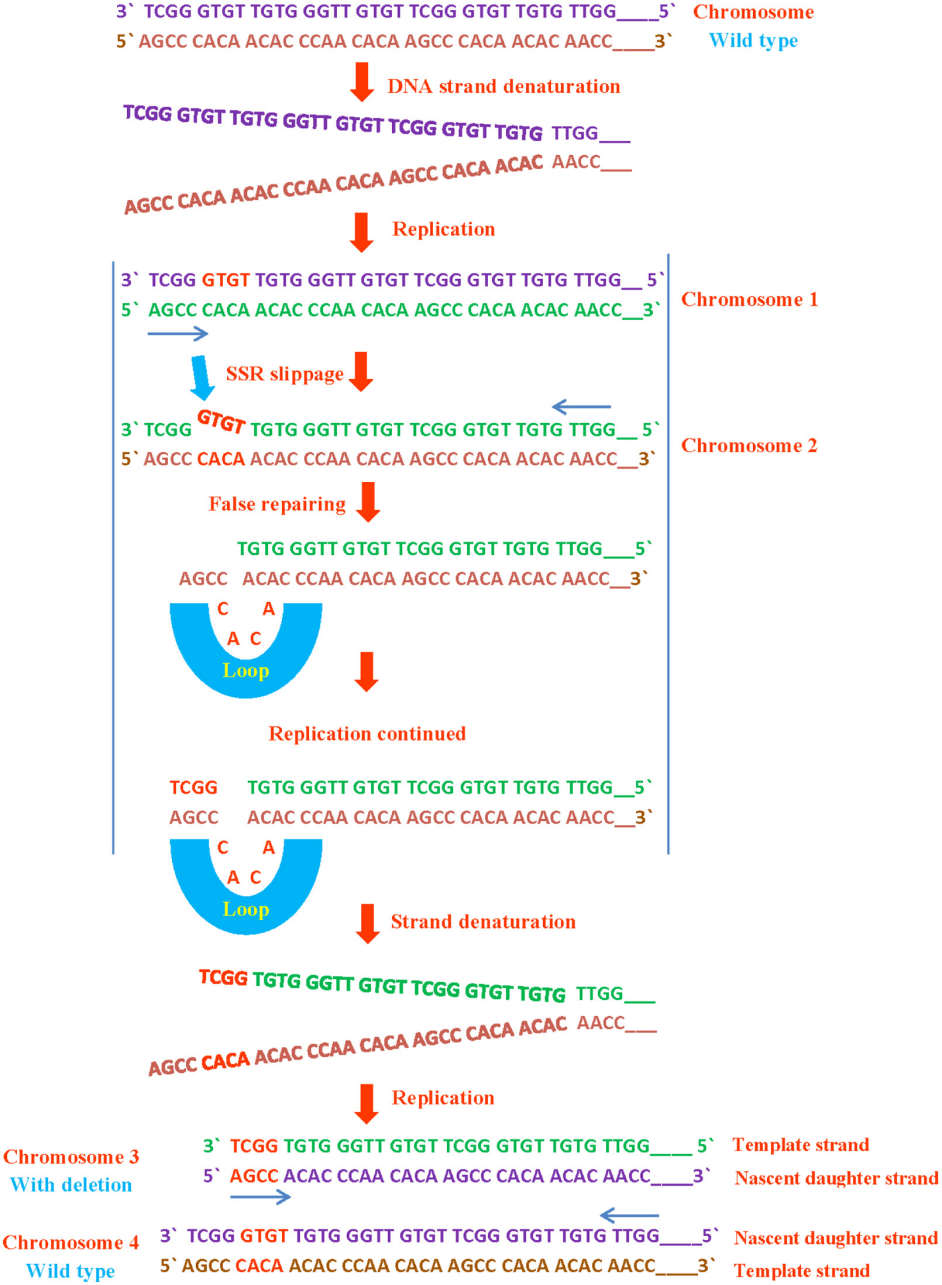
**SSR deletion during DNA replication.** If a SSR slips or loops out from template strand, it results in deletion. These mutations cause detrimental effects on normal protein function due to replacement of amino acid as has been seen in various microbes following genomic evolution.

**FIGURE 3:**
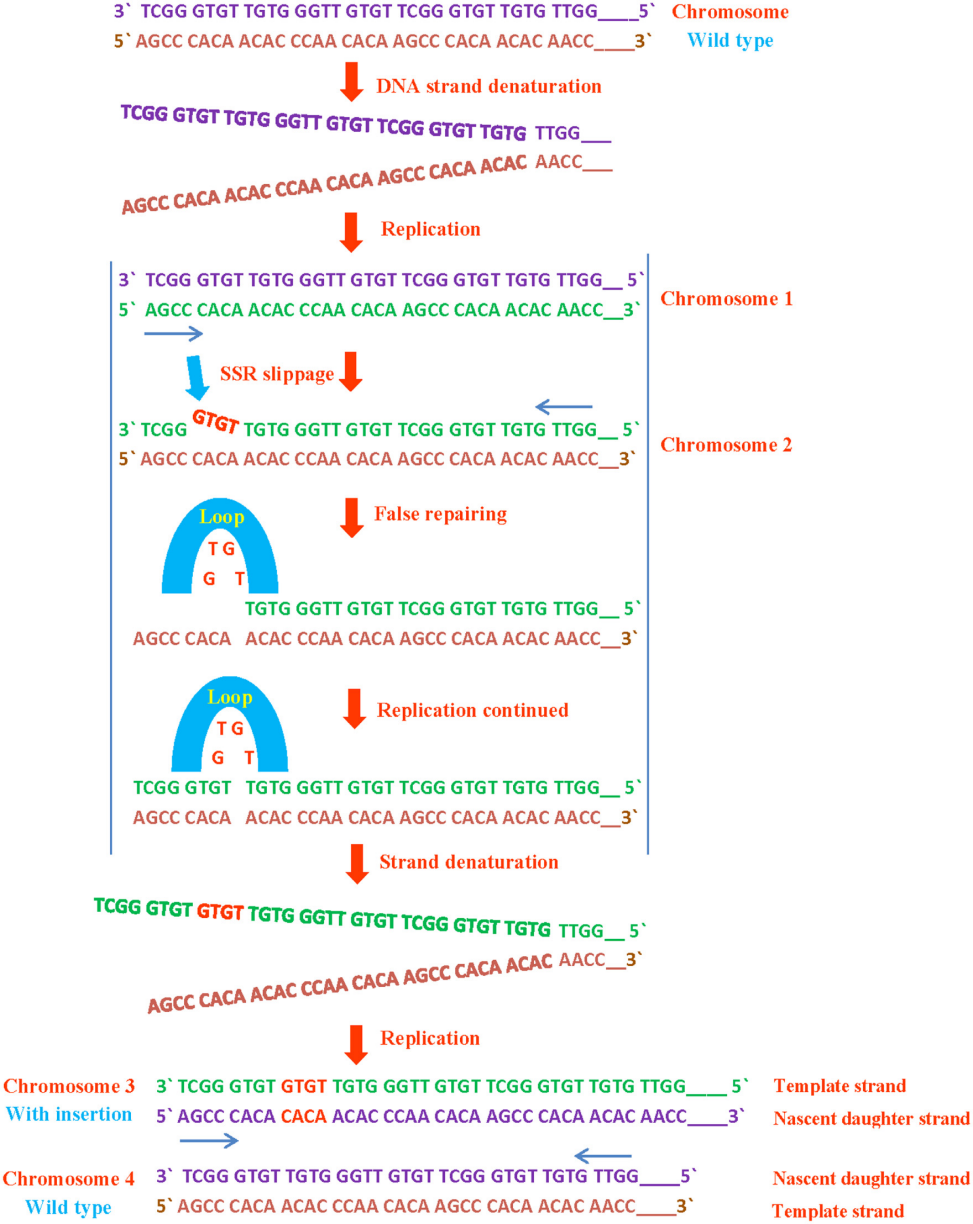
**SSR insertion during DNA replication.** If a SSR slips or loops out backward from template strand, it rearranges and inserted in form of duplication or at other site in template strand which mutates the normal sequence leading to the translational and translational mutations.

SSRs are susceptible to replication mispairing slippage. Slippage involves a region of non-pairing (shown as a loop) containing backward or forward slippage loop repeats of nascent daughter strand or of the parental strand, results in an insertion or a deletion on both strands respectively (**Figures 2** and **3**). Subsequently, it is possible that slipped strand mispairing can also cause insertions/deletions in non-replicating DNA. In such cases, non-pairing is occurred in two regions of repeats positioned on both complementary DNA strands (Levinson and Gutman, 1987). The replication slippage predicts persistent deletions, duplications and insertions at infinite degree between non-contiguous repeats; this type of slippage is a leading cause of genomic evolution (Dover, 1995).

### Sequence Mutations and Evolutionary Changes in Microbes

Microsatellites have been produced a vast number of amino acid repeat sequences in roughly 20 to 40% proteins found in various genomes (Marcotte et al., 1998). These repeats occur at protein coding sites in a genome and consist of trinucleotides (Sutherland and Richards, 1995). In yeast, these sequences are transcribed repeatedly in same amino acids such as glutamine, glutamic acid, asparagine, aspartic acid and serine affecting physical and chemical properties of the proteins. Such variations gradually modify the normal protein functions (Hancock and Santibáñez-Koref, 1998). The mutation rate measured for average microsatellite loci was 2.97 × 10^-4^ observed in yeast *Aspergillus fumigatus*. Yeast genome contains large number of microsatellites to offer targets for direct investigation (Strand et al., 1994, 1995; Séré et al., 2014).

Length mutations in FLO1 gene regulate the adhesive properties in bacterial membrane. These sequence mutations provide evolutionary modifications to the membrane surface proteins. Consequently, varying the adhesive features which assist pathogenic microbes to resist immunological changes in the hosts (Moxon et al., 1994; Verstrepen et al., 2005). Michael et al. (2008) reported length variations in microsatellites in fungus (*Neurospora crassa*); these variations control the time length of circadian clock cycle. Unwanted evolution induced by microsatellite deletions and indels can rapidly decline the performance of genetically engineered circuits and metabolic pathways in microbes (Jack et al., 2015).

### Gene Regulation by Sequence Mutation

Fimbriae formation in *Haemophilus influenza* is stimulated by unit mutations in microsatellite sequences by the modification of promoter spacing (Moxon et al., 1994). Microsatellites cause mutation instability in colorectal cancer infected with viruses (Rooney et al., 2014) by altering splicing or gene regulation. This includes nucleotide variations projected to cause missense swaps, small in-frame insertions/deletions or intragenic/intergenic sequence (Thompson et al., 2014; Thompson and Spurdle, 2015). SSRs have been accountable for the phase changes with the support of variation in promoter activity and gene transcription (Van Ham et al., 1993; Dawid et al., 1999; Martin et al., 2005). Oligopurine/oligopyrimidine with long tracts was discovered in bacterial genomes near regulatory regions (Holder et al., 2015).

## Disadvantages and Limitations in Microsatellites Analysis

Currently, molecular markers are very expensive for most wide-ranging applications; they have weaknesses in sequence determination, sequence information, unsuitable across species, numerous bands per reaction and misinterpretation in terms of loci and alleles (Miah et al., 2013). Due to the limited availability of genomic sequences of prokaryotic species at various genomic databases, it is not easy to analyze microsatellite sequences in vast reaches of DNA. Sometimes, microsatellite loci are not accommodating in determining the evolutionary relationships in distantly related species (Barbará et al., 2007), so in order to evaluate the occurrence of repeats for their identification and *de novo* characterization in individual genomes, massive degree of time duration and expensive research work is needed. The key drawback is that, microsatellites are isolated *de novo* from the species studied first time (Zane et al., 2002). Because of two main facts: (i) They are located in non-coding regions with higher rate of nucleotide substitution compared to the coding regions. Therefore, it is problematic to design universal primers corresponding conserved sequences. (ii) When engaging the identical primer pair, nucleotide switches within the repeats are observed between species (Clisson et al., 2000).

Therefore, study and construct of unknown microsatellites clone libraries depends on the occurrence of particular SSRs in genomes of interest (Zane et al., 2002; Selkoe and Toonen, 2006). The occurrence of microsatellites reported in various microbes employed in molecular studies is significantly different (Tóth et al., 2000). Sometimes, it has been documented that it is extremely difficult to obtain microsatellites and other repeat sequences from a particular DNA sequence (Dutech et al., 2007).

## Conclusion

Microsatellites and their significance in determination and understanding microbial genome evolution have been established in present study. Microsatellites are important evolutionary markers which are useful in tracking SSRs length variations such as point mutations, duplications, DNA repair, and replication slippage in phyletic lineages stretched across the entire genomes. Additionally, novel SSR analysis techniques and sequencing methods are discussed in this study, which are useful for the determination of potent evolutionary markers for previously deserted microbial genomes. Microsatellite repetitions can be traced by pursuing these advanced sequences techniques and more refined research databases. This review will highlight new insights into these biologically active and significant marker tools for studying genomic evolutions in future research and will also extend further investigations on microsatellites and other sequence repeats in the field of microbiology.

## Conflict of Interest Statement

The authors declare that the research was conducted in the absence of any commercial or financial relationships that could be construed as a potential conflict of interest.
